# Supplementary material for: All-water supercapacitor enabled by 1-nm clay channels
Source: Nat Commun. 2026 Jun 5;17:5014. doi: 10.1038/s41467-026-73924-1 (PMC13241519; doi:10.1038/s41467-026-73924-1)
Supplement: Supplementary file 1 — Supplementary Information [file 41467_2026_73924_MOESM1_ESM.pdf]

Supplementary Information

All-water supercapacitor enabled by 1-nm clay channels

by

Vasily Artemov*, Svetlana Babiy, Yunfei Teng, Jiaming Ma, Alexander Ryzhov, Tzu-Heng Chen, Lucie Navratilova, Victor Boureau, Pascal Schouwink, Mariia Liseanskaia, Patrick Huber, Fikile Brushett, Lyesse Laloui, Giulia Tagliabue, Aleksandra Radenovic

*Corresponding author: Vasily Artemov; Email: vasily.artemov@tuhh.de

This PDF file includes:

1. Clays: abundance, atomic structure, physical and chemical properties 2
2. Protocol for the raw materials cleaning 7
3. Atomic composition of membrane-electrode unit 8
4. Clays' electrical properties 10
5. 'Ionizing' behavior of clay 13
6. Blue capacitor electric properties 15
7. SEM, TEM, and STEM imaging 26
SI References 32

Including:

Figures S1 to S36
Tables S1 to S3
SI Reference 1-10

1. Clays: abundance, atomic structure, physical and chemical properties

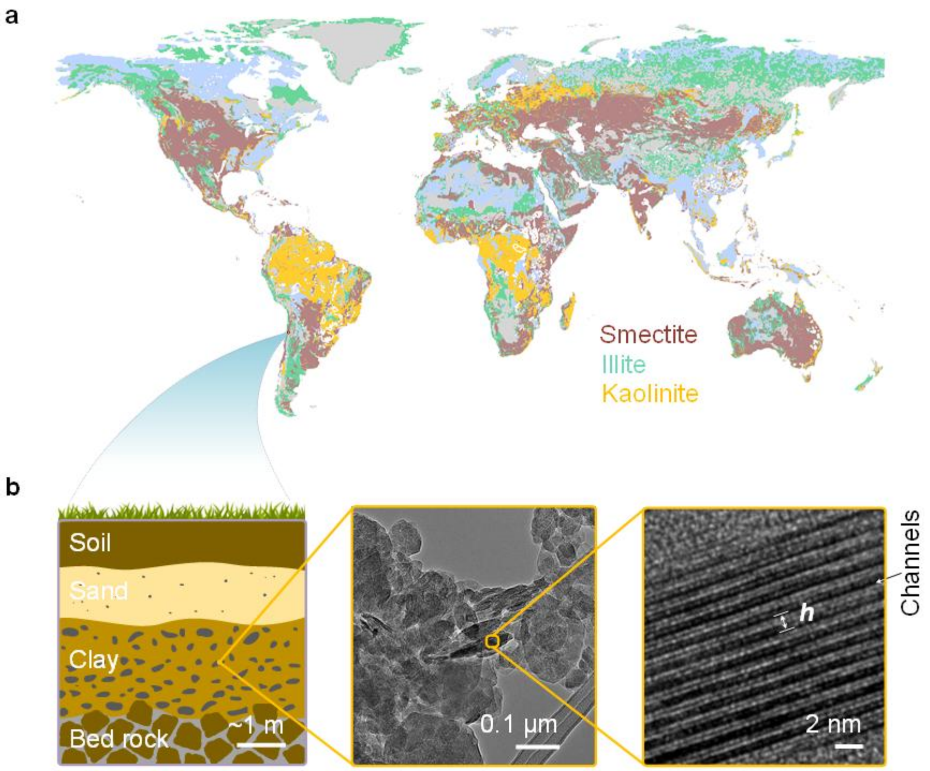

Figure S1. (a) Topsoil clay distribution map (data [1]) depicting regions with areal density of clay exceeding 10 kg/m^2: smectite (brown), Illite (green), kaolinite (yellow). (b) Multi-scale images of clay: bulk soil cross-section (left), aggregated sheets in TEM (middle), and nanosheet texture in high-resolution TEM (right).

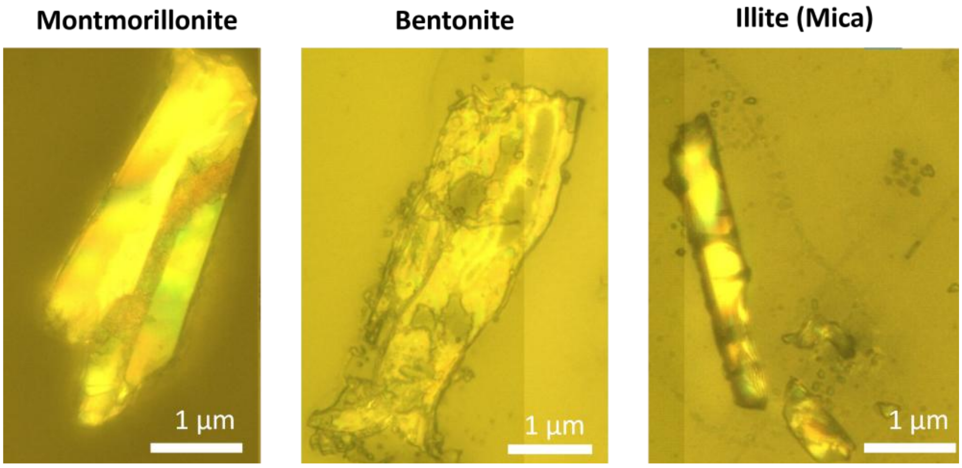

Figure S2. Optical photos of large clay crystals of montmorillonite, bentonite, and illite, from left to right, respectively.

Table S1. ξ-potentials (in mV) of the nano-colloidal solutions of different clay types and graphene in pure 18-MΩ water with a pH of 7. Measurements were done with Malvern Panalytical™ Zetasizer.

Meas. number	Smectite (MMT)	Kaolinite	Illite	Graphene
1	-30.9	-17.2	-15.2	-16.2
2	-31.7	-16.4	-16.6	-15.5
3	-30.6	-16.7	-15.2	-14.9
Average	-31.1	-16.8	-15.7	-15.5

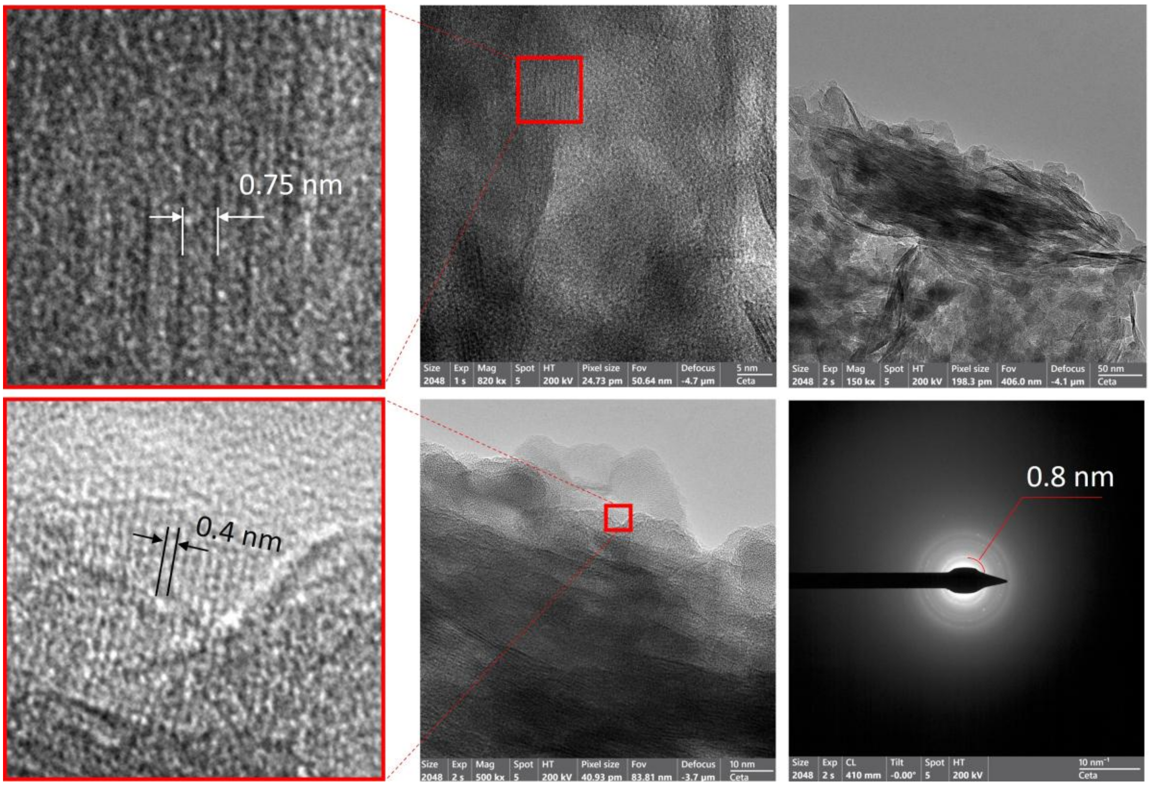

Figure S3. Transmission electron microscopy (TEM) images and selected area electron diffraction (SAED) pattern of montmorillonite (MMT) crystals.

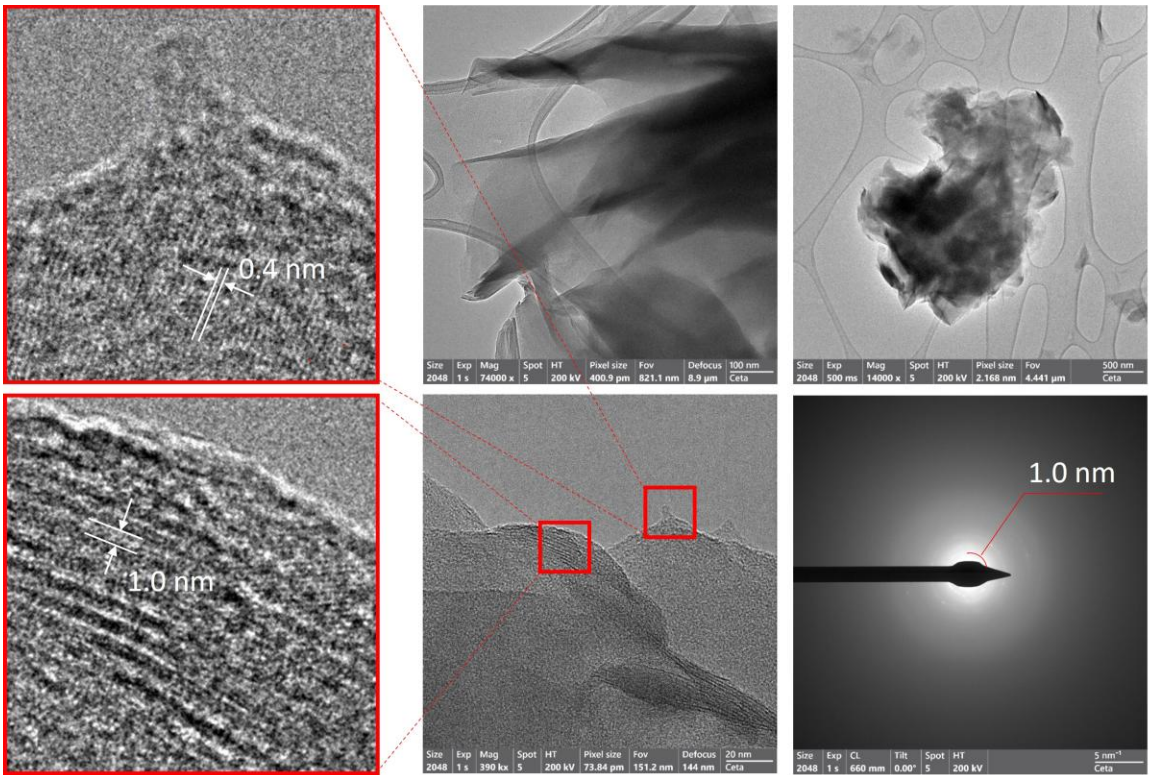

Fig. S4. The same as in Fig. S3 but for bentonite crystals.

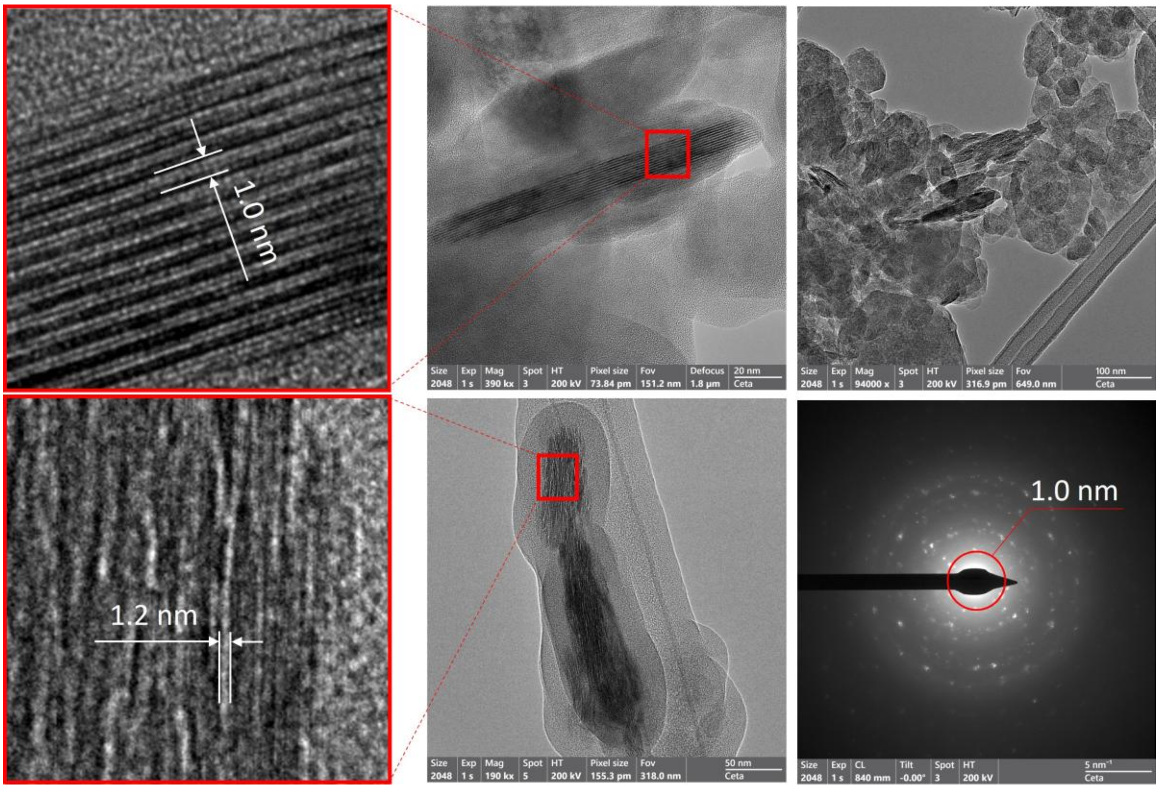

Figure S5. The same as in Fig. S3 and S4 but for illite crystals.

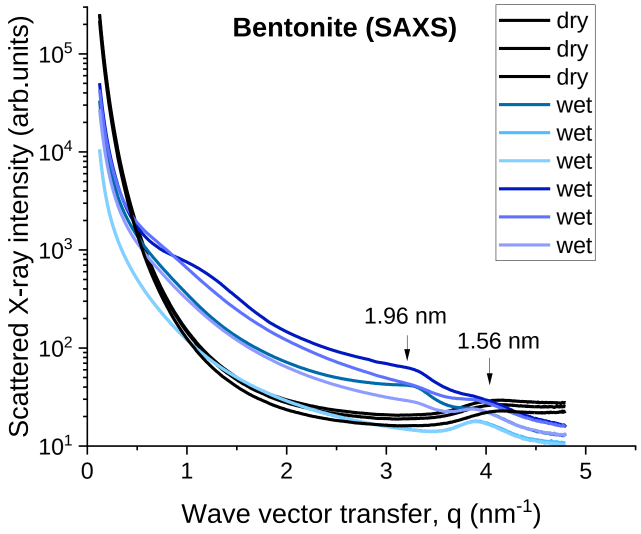

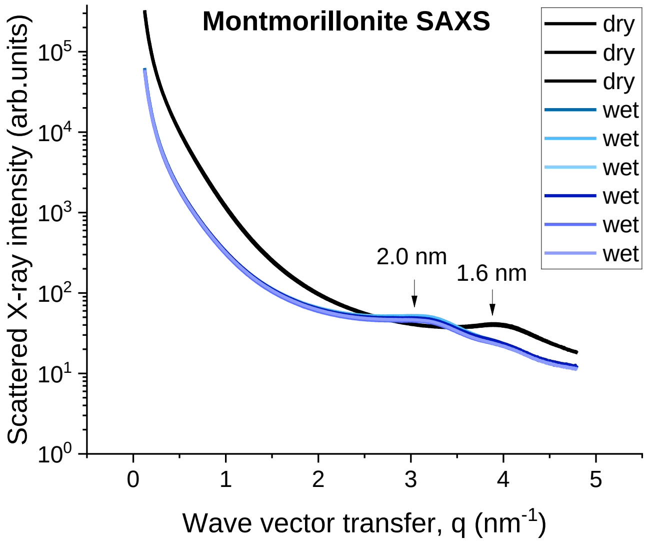

Figure S6. Small-angle X-ray scattering (SAXS) of wet and dry bentonite (top) and montmorillonite (bottom) clay powders, recorded at the P62 SAXS/WAXS beamline of the PETRA III synchrotron source. The numbers next to the arrows indicate the interlayer distances corresponding to the peak maxima in reciprocal space. Note that the semi-dry sample was stored in a low-humidity environment without vacuum or annealing, resulting in a larger interlayer distance compared to the vacuum-dried TEM samples in Figs. S3 and S4. Source data are provided as a Source Data file.

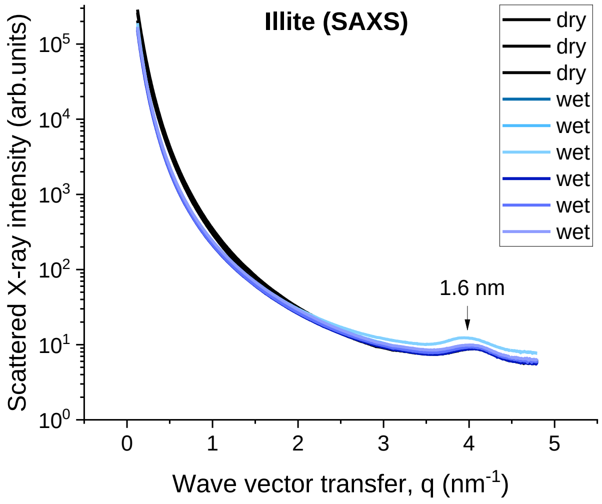

Figure S7. Same as in Fig. S6 for illite clay powders. No diffraction peak shift indicates no water penetration between the crystalline layers (see Fig. 2c of the main text). Source data are provided as a Source Data file.

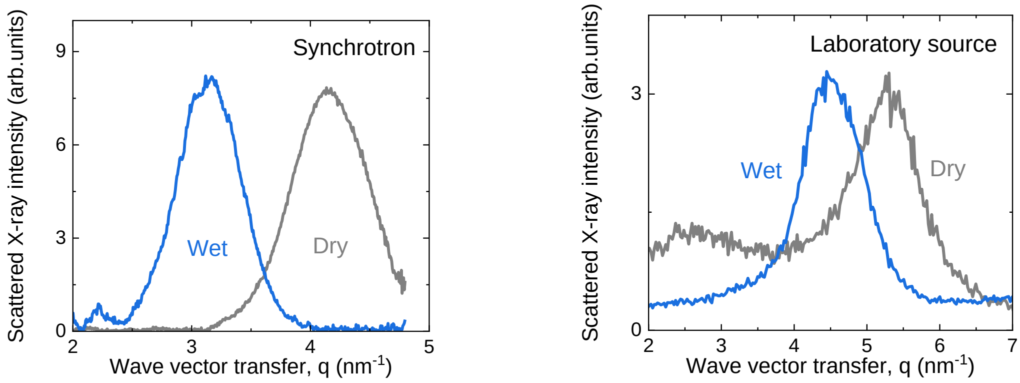

Figure S8. Small-angle X-ray scattering (SAXS) of wet and dry bentonite clay powders. The data show the first diffraction peak. The graph on the left is synchrotron data (Fig. S6 with subtracted baseline). The samples were immersed in liquid water. The data on the right is Bruker XRD laboratory source measurements. The samples were prepared in saturated water vapor. The shift of the peaks in both measurements confirms water penetration between the bentonite clay crystal layers. Source data are provided as a Source Data file.

2. Protocol for the raw materials cleaning

The clay treatment protocol closely follows well-established methodologies reported in the literature [2-8]. The primary objective of the cleaning procedure is to remove mobile cations that could affect electrical conductivity.
The exact cleaning steps used in our study are as follows:

a) **Suspension preparation:** Approximately 50 g of raw clay powder was dispersed in 3 liters of distilled water using magnetic stirring combined with ultrasonic homogenization. By comparison in [3], a higher concentration of 100 g L^{-1} was used.

b) **Sedimentation:** The suspension was left undisturbed for two days to allow natural precipitation, resulting in size-based fractionation of particles.

c) **Top fraction selection:** The fine particle fraction at the suspension surface was carefully extracted by automated pipetting.

d) **Multiple centrifugation:** The extracted supernatant underwent centrifugation at 8000 rpm to separate sediment. This speed is higher than that used in [3] (3500 rpm). Following each centrifugation, the supernatant was replaced with distilled water, and the sediment was resuspended using stirring and sonication. This cycle was repeated ten times.

e) **Conductivity control:** The conductivity of the supernatant was monitored throughout the centrifugation cycles (Fig. S9). Initially, it was approximately 0.1 S cm^{-1} due to dissolved surface cations. Conductivity progressively decreased, reaching a plateau near 1 μS cm^{-1} around the seventh cycle, indicative of pure water under laboratory conditions. Similar values have been reported in [4] following comparable cleaning.

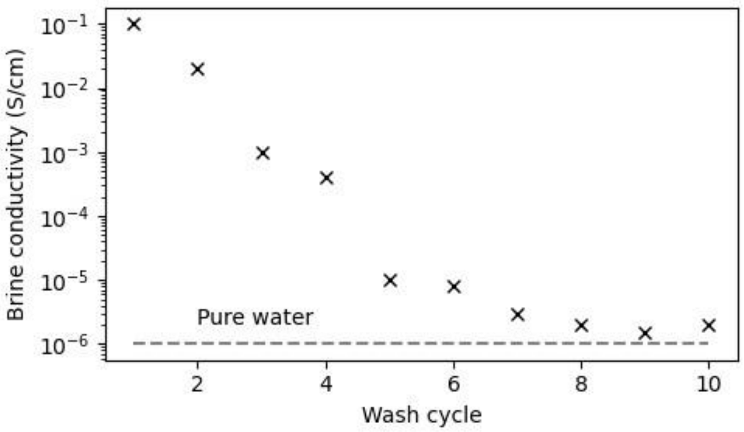

Figure S9. Electrical conductivity of the brine at different cycles of the clay washing via centrifugation at 298 ± 1 K. The saturation level corresponds to the conduction of pure water in the laboratory atmosphere. Source data are provided as a Source Data file.

f) **pH control.** The supernatant pH was periodically checked, stabilizing near neutral

g) **Dialysis:** To further purify the suspension, osmotic dialysis was performed on the supernatant after the tenth centrifugation cycle, following procedures similar to [4]. Dialysis occurred over approximately one week with daily water changes.

h) **Freeze-drying:** The dialyzed suspension was freeze-dried to produce dry clay powder, which was subsequently milled to homogeneity and used for device fabrication.

i) **Atomic composition control:** Energy-dispersive X-ray spectroscopy (EDX) analysis comparing pristine clay to device-level materials (Fig. S11) shows that Na and K peaks present in raw clay are absent after cleaning. This is further corroborated by elemental mapping of device cross sections (Fig. S10).

3. Atomic composition of membrane-electrode unit

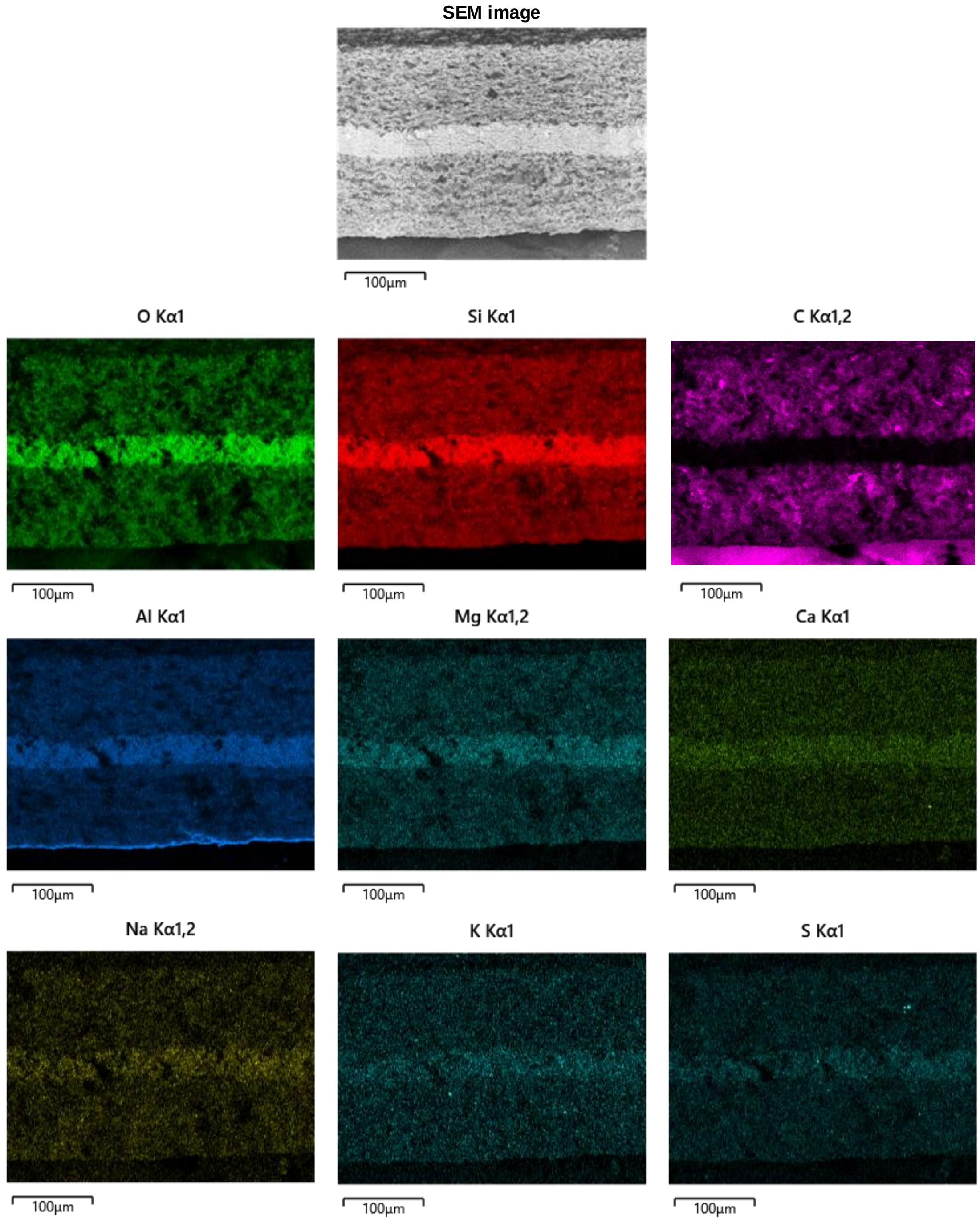

Figure S10. SEM energy dispersive X-ray (EDX) element mapping for a cross-section of a membrane-electrode unit (MEU) made of smectite clay and graphene. Color maps are associated with the chemical species expected in the system (O - green, Si - red, C - magenta, Al - blue, and Mg - cyan), in agreement with crystal structure, plus traces of Ca – dark green, Na - yellow, K, and S – light blue. The top is the SEM micrograph. The EDX sum spectrum of the element mapping is shown in Fig. S11.

a

b

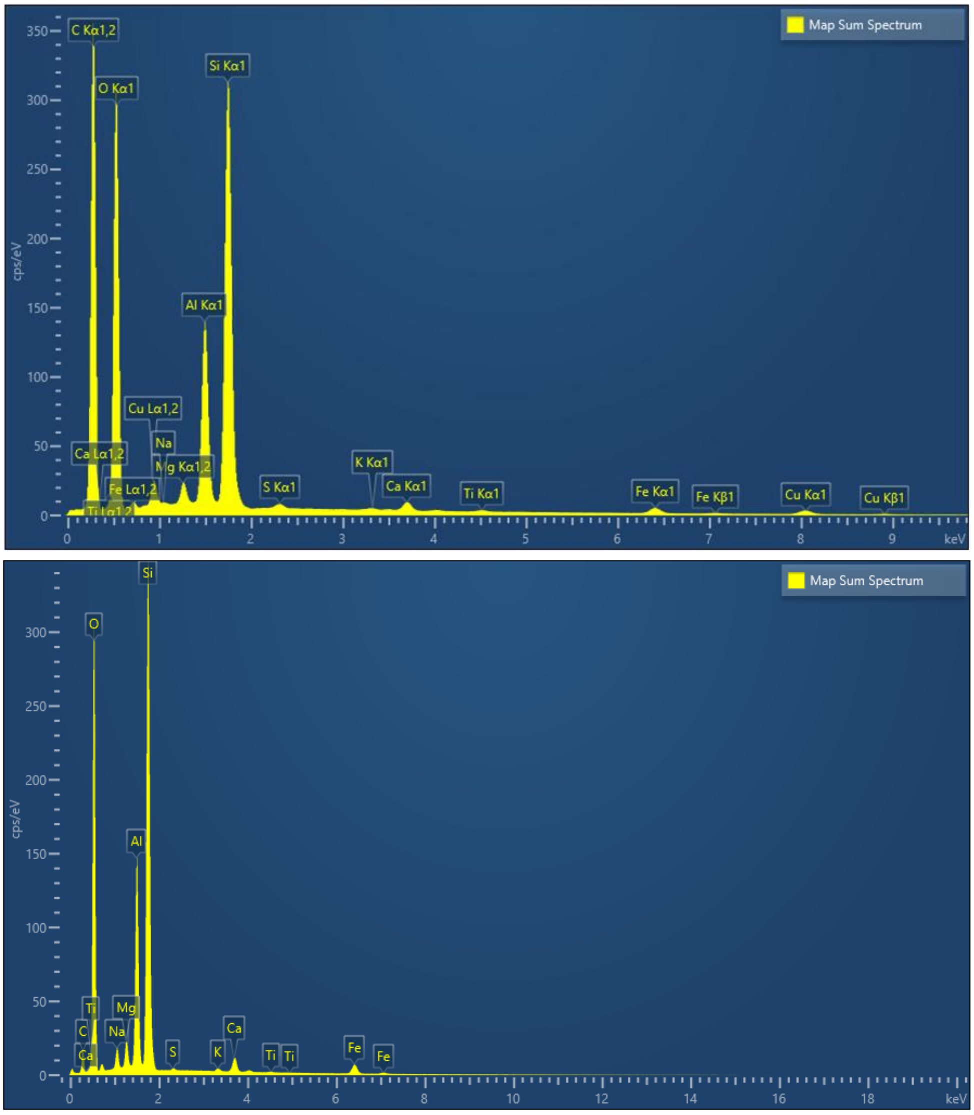

Figure S11. Contents of clay before and after washing. (a) EDX sum spectrum of the elements constituting the device of this study associated with the elemental map shown in Fig. S10. (b) EDX sum spectrum of the pristine clays.

4. Clays' electrical properties

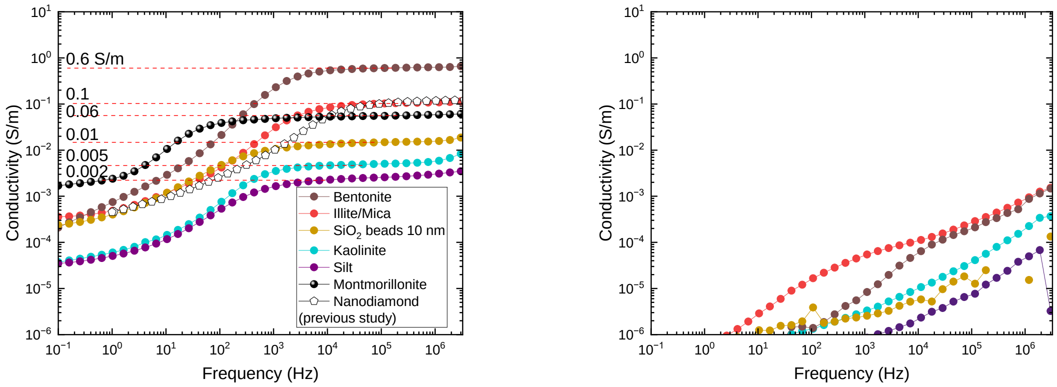

Figure S12. Proton conductivity of compacted clays on water content. (left) Spectra of proton conductivity in different clay materials compared with compacted SiO_2 beads of 10 nm, and 5-nm nano-diamond ceramics (data from [9]). (right) The same spectra of clays, but after annealing at 393 K for 6 hours. All data obtained at 298 ± 1 K. Note, the dry samples were filled with water by long exposure to saturated water vapor, excluding contamination. Source data are provided as a Source Data file.

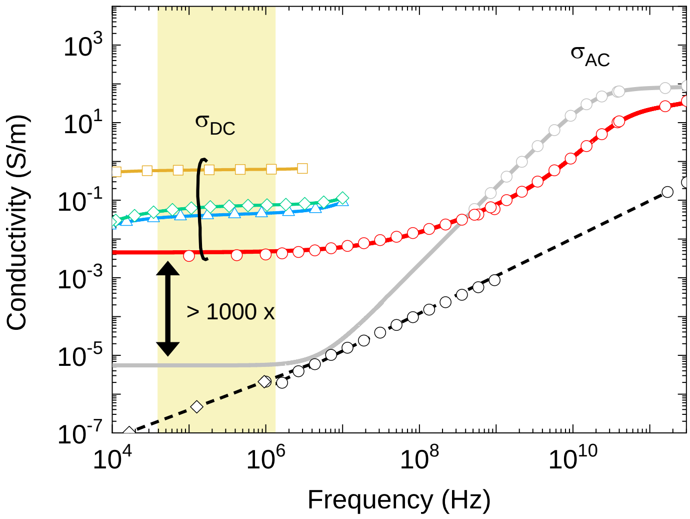

Figure S13. Electrical conductivity spectra (log-log scale) for various water-impregnated materials: MCM-41 (red, data adapted from [10], pore sizes 3–9 nm), Nafion (blue, our data, 4–20 nm pores), nanodiamond ceramics (green, adapted from [9], 5 nm pores), and nanostructured clay (orange, this study, 1 nm pores). Bulk water data shown in gray; dry materials' spectra in black. Yellow stripe indicates the region where the DC conductivity is assessed. All data obtained at 298 ± 1 K. Source data are provided as a Source Data file.

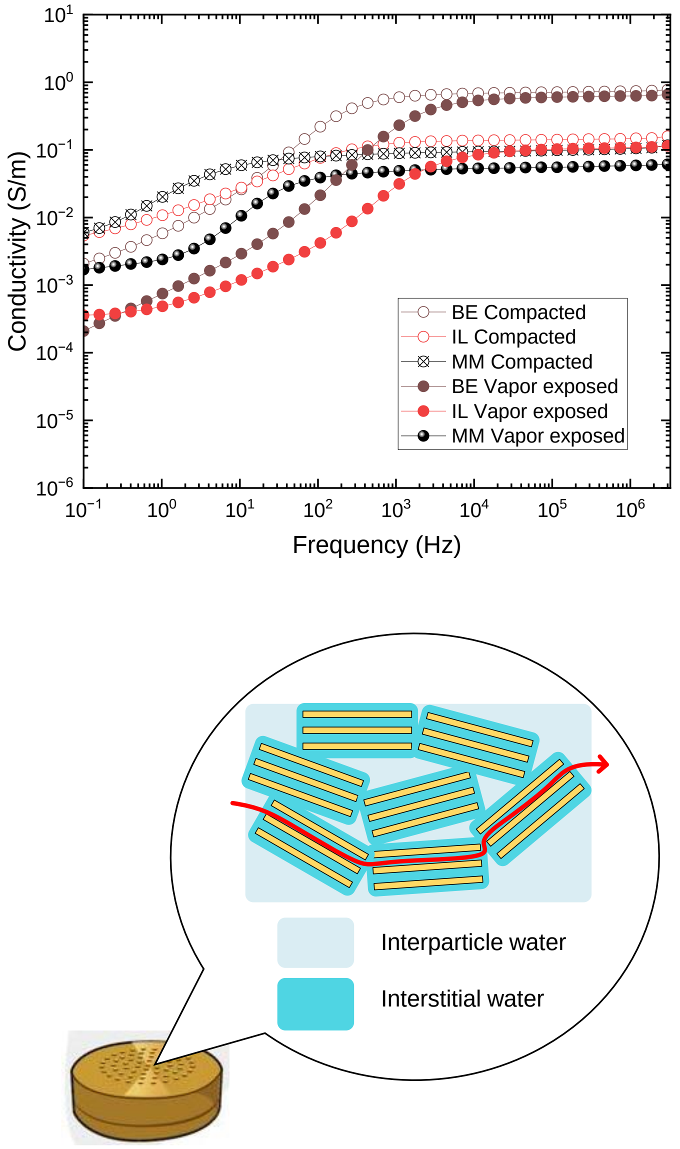

Figure S14. Proton conductivity of clays in micro and nanopores. Comparison of electrical (proton) conductivity of the saturated-water-vapor-exposed clay samples (bentonite, illite, and montmorillonite) with that placed in liquid water under high pressure. All data obtained at 298 ± 1 K. The bottom picture shows the difference between the interstitial water between the clay crystal layers and the interparticle water in micropores between the clay particles. A minor difference between the two cases indicates that the conductivity originates from the nanopores rather than large micropores. Source data are provided as a Source Data file.

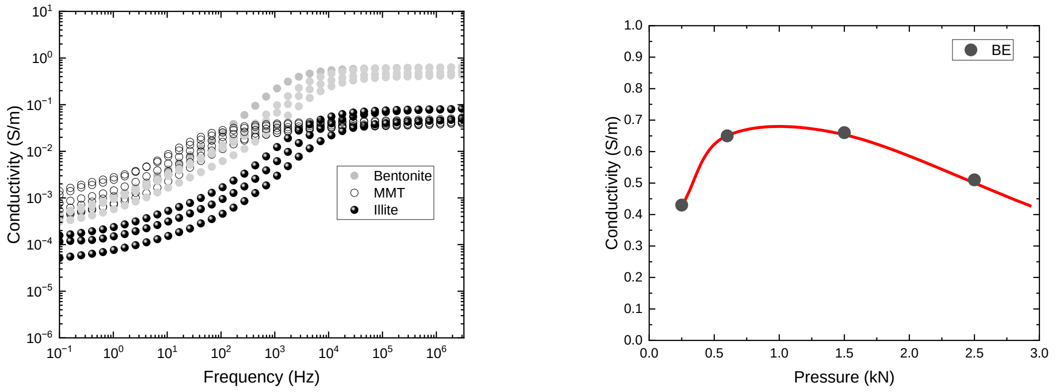

Figure S15. Proton conductivity of clays depending on the applied pressure. The left graph shows the measurement of electrical conductivity at different pressures at 298 ± 1 K. The right graph shows the dependence of the DC conductivity plateau for bentonite clay. The red-curve fit shows that the pressure dependence of the clay proton conduction is weak. This confirms the results shown in Fig. S14 because pressure affects the large pores rather than the nanopores. Source data are provided as a Source Data file.

5. ‘Ionizing’ behavior of clay

As demonstrated above, the washed clays do not release foreign ions directly into the water, as shown by the unchanged conductivity of the supernatant (Fig. S9). However, they influence the self-ionization equilibrium of the water layers adjacent to clay surfaces (main text Fig. 6), leading to the generation of excess protons.

To evaluate the impact of this effect on proton conductivity, we prepared suspensions of washed clay powders in deionized water at concentrations spanning from 10 µM to 0.1 M. Suspensions were homogenized using a high-power ultrasonic device. Electrical conductivity measurements were performed over a frequency range of 0.1 Hz to 0.1 MHz (Fig. S16) using a Teflon measurement cell equipped with approximately 2 cm^2 gold electrodes spaced 3 mm apart.

At low clay concentrations (below 0.02 M), no significant influence of colloidal particles on conductivity was observed (Fig. S16b), likely because the average interparticle distance greatly exceeds individual particle sizes. However, at concentrations above 0.02 M, conductivity exhibited an exponential increase with a factor approximately 0.65, close to 2/3, correlating with the total surface area of the particles.

The limiting case of this trend is represented by the electrical conductivity of water confined within the 1-nm channels of solid clay matrices, which exceeds the measured range.

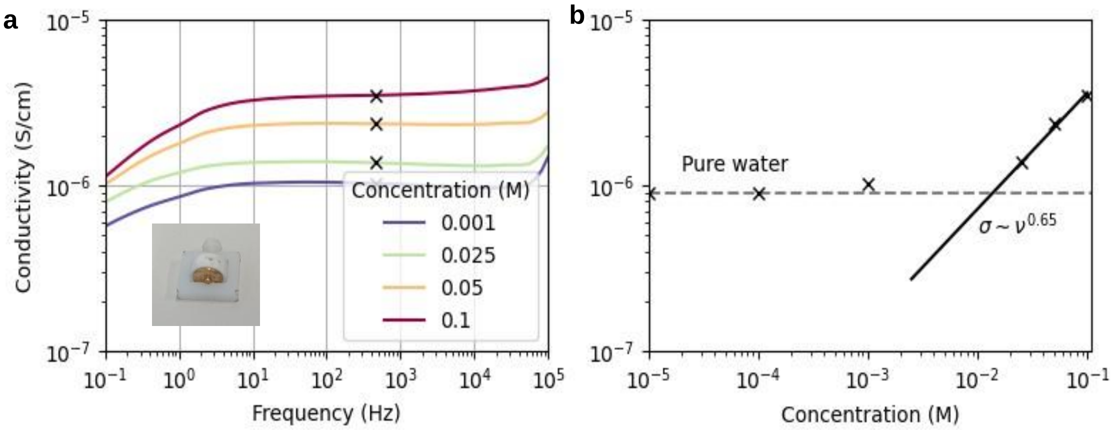

Figure S16. Clay suspensions conductivity. (a) Conductivity spectra of colloidal suspensions of montmorillonite at varying concentrations (see the legend) at 298 ± 1 K. Crosses denote DC conductivity plateaus. Inset shows the measuring cell of 5 mm in diameter. (b) DC conductivity vs. clay concentration. The dashed line indicates the conductivity of deionized water under laboratory conditions. Source data are provided as a Source Data file.

Note, electrical conductivity σ in nanoparticle systems follows concentration-dependent scaling: $\sigma \sim \nu^{\alpha}$, with α determined by the percolation regime. In pre-percolation / near-percolation regime α < 1 (including 2/3), the conductivity is limited by aggregation, Brownian motion, and electrostatic effects, and transport pathways are incomplete. Above percolation threshold α = 1.7 ± 0.2, continuous conductive network is formed, and exponent depends on particle geometry (elongated particles give smaller α). Finally, in saturation regime (clay membranes), conductive pathways are already established, and σ becomes weakly dependent on concentration.

Thus, the ionizing behavior of clay manifests through enhanced proton activity within a 1.0 ± 0.1 nm interfacial water layer, where electrostatic interactions surpass thermal energy (k_BT). At low clay concentrations this effect is negligible, but becomes significant as clay particles approach and interfacial water layers overlap, leading to a proportional increase in proton conductivity relative to particle surface area.

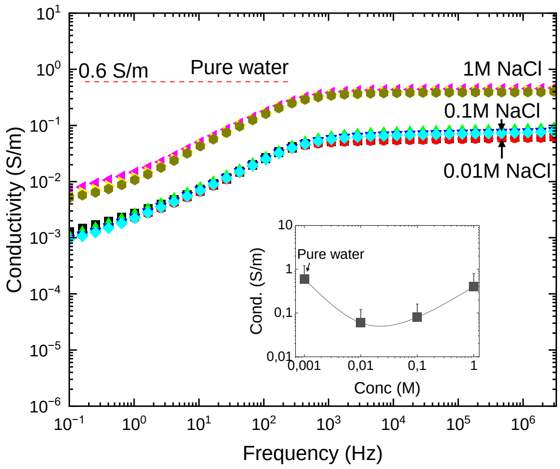

Figure S17. Clay conductivity on salt concentration. Conductivity spectra of clay membranes impregnated with NaCl solutions at different concentrations at 298 ± 1 K. The dashed red line indicates the electrical conductivity of clay containing pure water measured earlier. The inset displays DC conductivity plateau dependence on concentration; the pure confined water data point is shifted to 0.001 M for clarity. Data presented as mean +/- SD (n=3 technical replicates). Source data are provided as a Source Data file.

6. Blue capacitor electric properties

We characterized the cell using cyclic voltammetry (CV) and charge-discharge (CD) tests. In the CV measurements, the current was monitored while varying the voltage at a fixed rate. In contrast, during the CD tests, the voltage was adjusted while maintaining a constant current. CV measurements were employed to calculate the capacitance, *C*, using the following equation:

$$C = \frac{\int idv}{2\mu m \Delta V}, \tag{S1}$$

where *i* and *v* are the current and potential in the CV test, *μ* is the scan rate in V/s, m is the mass of active materials in grams, *ΔV* is the voltage (potential) window during discharge in V, *I* is the constant discharge current in A, and *Δt* is the discharge time in seconds.

CD curves were used to calculate the specific energy, E, using the formula:

$$E = \int_{DC} \frac{IUdt}{m}, \tag{S2}$$

and the specific power, P, by:

$$P = \frac{E}{\Delta t}. \tag{S3}$$

The energy and coulombic efficiencies were determined from the CD and CV tests by dividing the output by the input energy and charge.

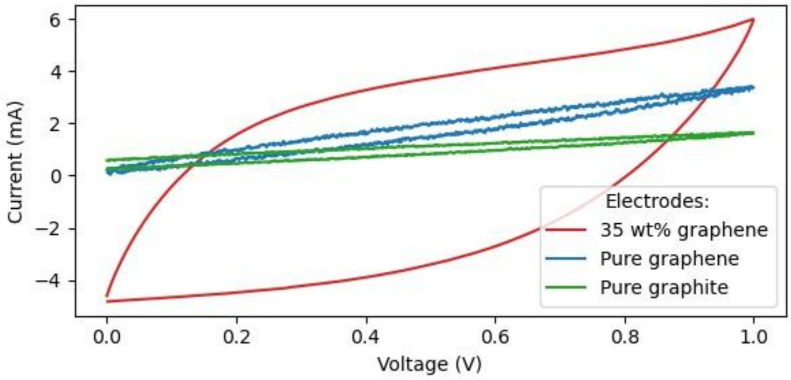

Figure S18. Different electrodes. Voltammograms comparing cells with fixed clay separator but electrodes of (a) 35% graphene-clay composite, (b) pure graphene, and (c) pure graphite at 298 ± 1 K. The composite electrodes (red curve) show superior capacitance with pure water as an electrolyte. Source data are provided as a Source Data file.

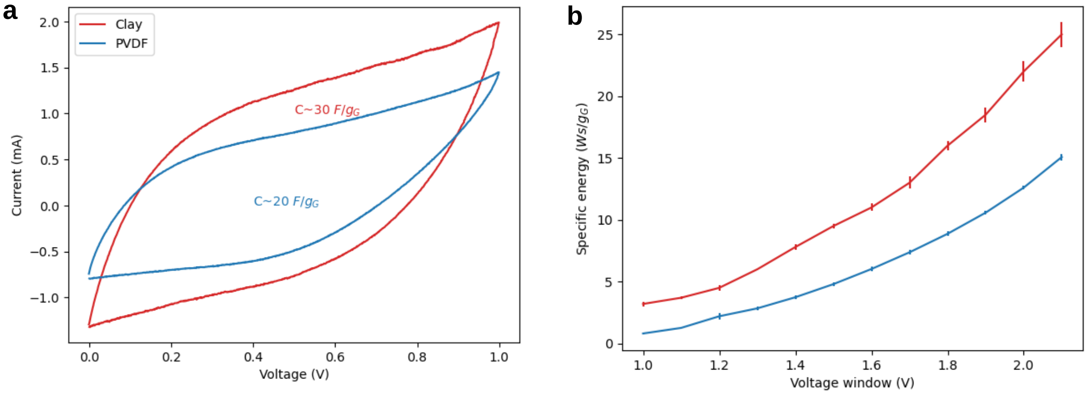

Figure S19. Different separators. (a) Cyclic voltammograms of two MEUs with the composite graphene-clay electrodes and a clay (red) and standard PVDF (blue) separators at scan rate 0.005 V/s. (b) Discharge energy at current 5 mA and different voltage windows normalized by graphene weight for the same MEUs. Data presented as mean ± SD (n=3 technical replicates). All data at 298 ± 1 K. Source data are provided as a Source Data file.

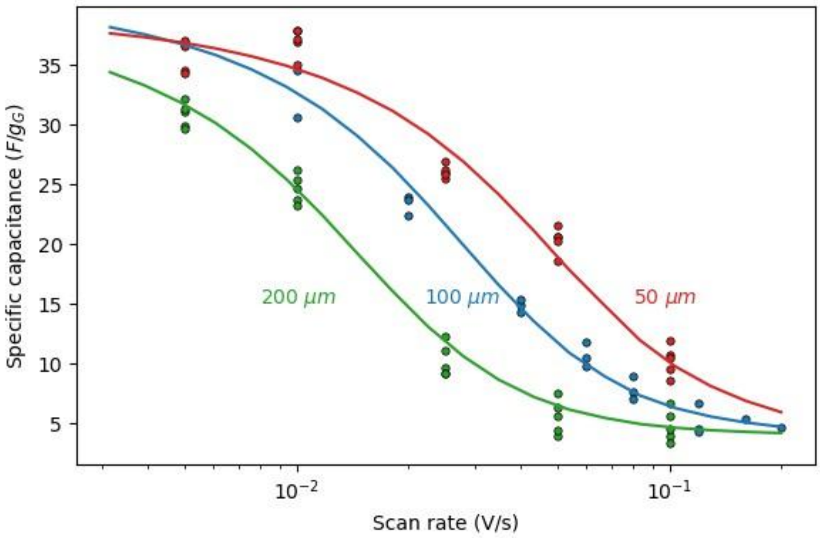

Figure S20. Different thickness of the clay separator. Gravimetric specific capacitance versus scan rate for MEUs with varying clay separator thicknesses at 298 ± 1 K. Experimental data (dots) and fits (curves). Source data are provided as a Source Data file.

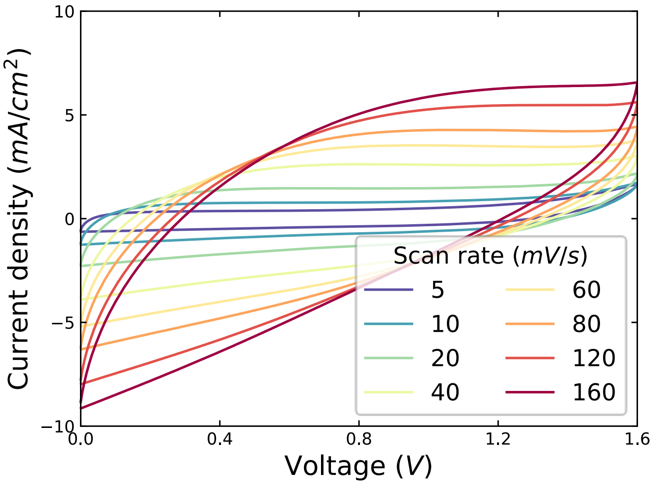

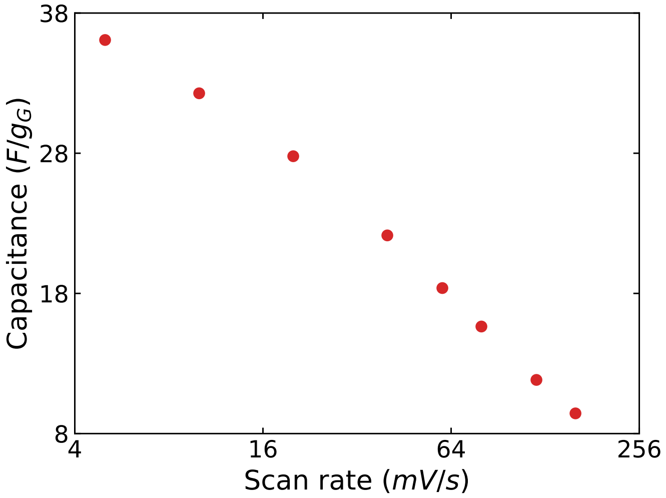

Figure S21. Different scan rates. Blue capacitor cyclic voltammograms (top) and specific capacitance vs. scan rate at 298 ± 1 K, calculated according to Eq. (S1). The voltage window is ΔV = 1.6 V. Source data are provided as a Source Data file.

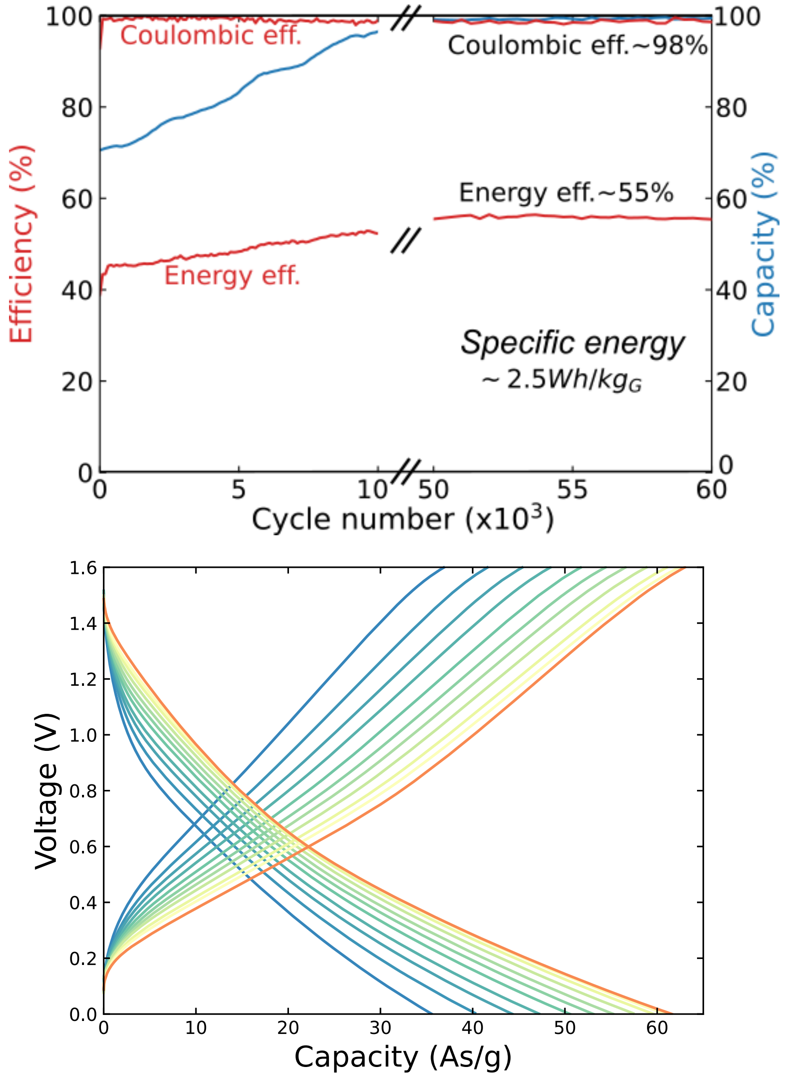

Figure S22. Long-term stability test. The figure shows the evolution of the blue capacitor's efficiency, capacity, and specific energy over more than 60,000 cycles at 298 ± 1 K. The initial period exhibits an "annealing" effect, likely due to the electrowetting of nanopores. The bottom graph displays the progression of the charge-discharge curves, with blue representing the initial cycles and red representing the final cycles. Source data are provided as a Source Data file.

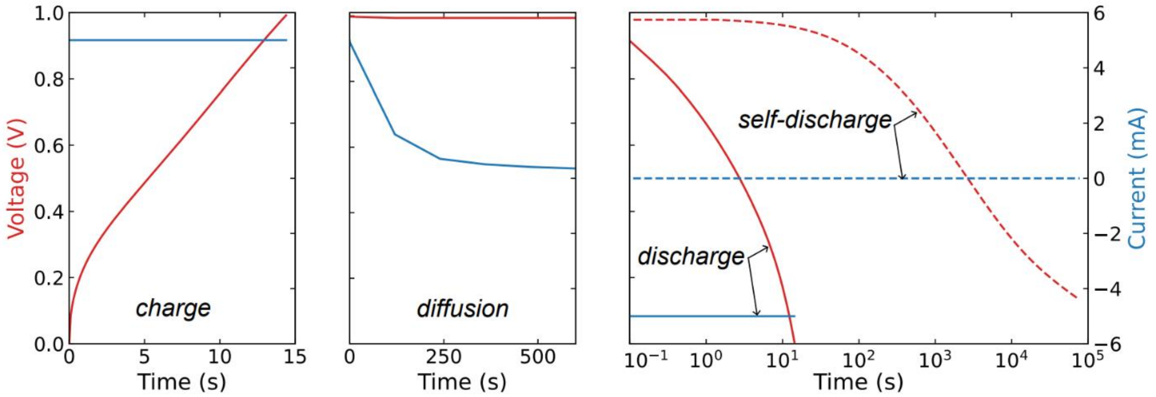

Figure S23. Self-discharge. The blue-capacitor charge phase with a fixed current (left), the diffusion phase with a variable current to maintain a constant voltage (middle), and the discharge phase (right), show both load discharge (solid line) and self-discharge with no load (dashed line). Note the logarithmic scale for the time in the right graph. The self-discharge occurs at a rate four orders of magnitude slower than the load discharge, indicating that the system is suitable for short- to mid-term electricity storage as a backup or an energy reserve, or a system where the fast charge-discharge and high power are needed, such as fast frequency control in the grid, or a regenerative brake system. All data at 298 ± 1 K. Source data are provided as a Source Data file.

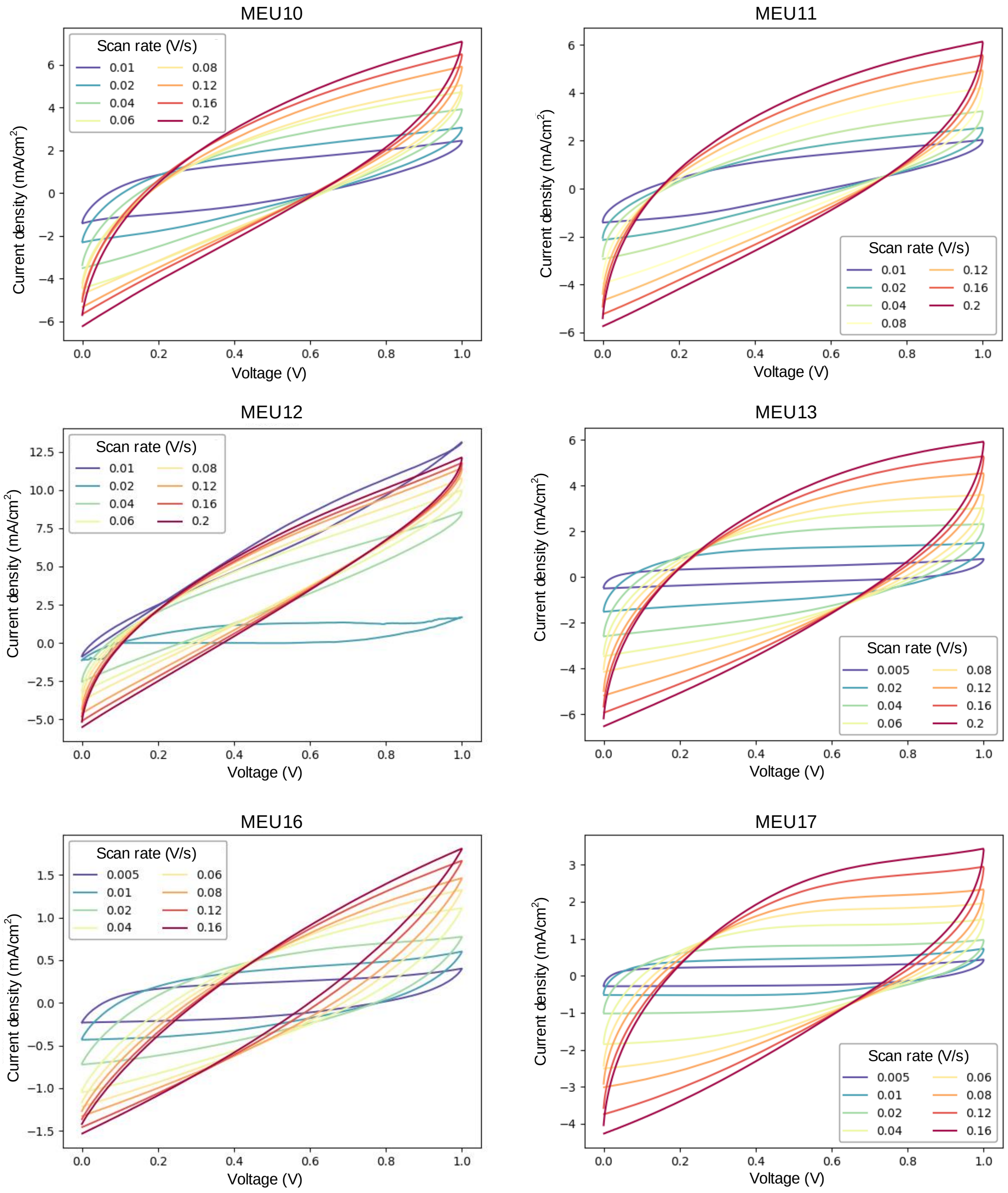

Figure S24. Cyclic voltammograms for several different MEUs (see the number on top of the panels) at different scan rates. For MEU composition, see Table S2. All data at 298 ± 1 K. Source data are provided as a Source Data file.

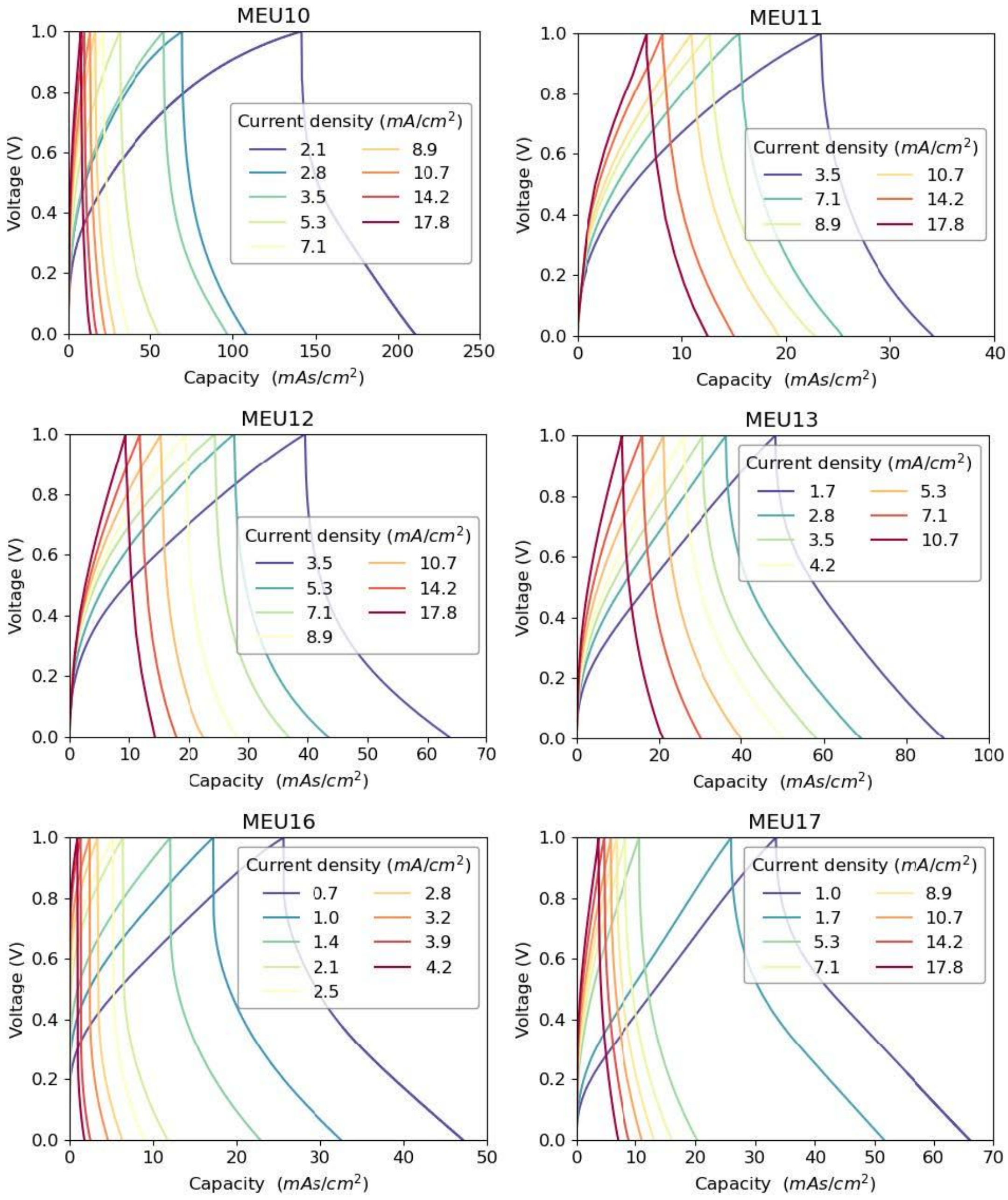

Figure S25. Charge-discharge curves for several different MEUs (see the number on top of the panels) at different current densities. For MEU composition, see Table S2. All data at 298 ± 1 K. Source data are provided as a Source Data file.

Table S2. The list of membrane-electrode units (MEU) produced and tested in this study.

MEU No	Electrodes	Separator	Sep. thickn. (μm)	Mass in the air (mg)	Wet mass (mg)	Mass gain (mg)
1	G 2mg/ml	BE 35 mg	15	39.9	42.6	2.7
2	G/BE 2ml/6mg	BE 35 mg	15	-	-	-
3	G/BE 1ml/10mg	BE 70 mg	30	87.8	92.6	4.8
4	G/BE 1ml/10mg	BE 35 mg	15	58	61.9	3.9
5	C/BE 5mg/5mg	BE 35 mg	15	-	-	-
6	G/BE/C 1ml/5mg/5mg	BE 70 mg	30	-	-	-
7	G/BE/C 1ml/10mg/5mg	BE 70 mg	30	103.3	110.2	6.9
8	G/BE/C 1ml/10mg/5mg	BE 35 mg	15	51.7	54.5	2.8
9	G/BE/C 1ml/10mg/5mg	BE 15 mg	7	34.8	38.2	3.4
10	G/BE 6ml/18mg	BE 35 mg	20	125.6	253.7	128.1
11	G/BE 10ml/18mg	BE 35 mg	20	91	619.5	528.5
12	G/BE 6ml/15mg	BE 15 mg	10	77	521.8	444.8
13	G/BE 6ml/15mg	BE 35 mg	20	77	195.5	118.5
14	C/G/BE 6ml/6ml/15mg	BE 35 mg	20	89	-	-
15	G/BE 6ml/15mg	BE 35 mg	20	77	-	-
16	G/BE 6ml/15mg	BE 35 mg	20	120	236.2	116.2
17	G/BE 6ml/15mg	BE 35 mg	20	120	289.5	169.5
18	G/BE 6ml/15mg	BE 35 mg	20	120	208	88

Note: G=graphene, BE=Bentonite (Smectite).

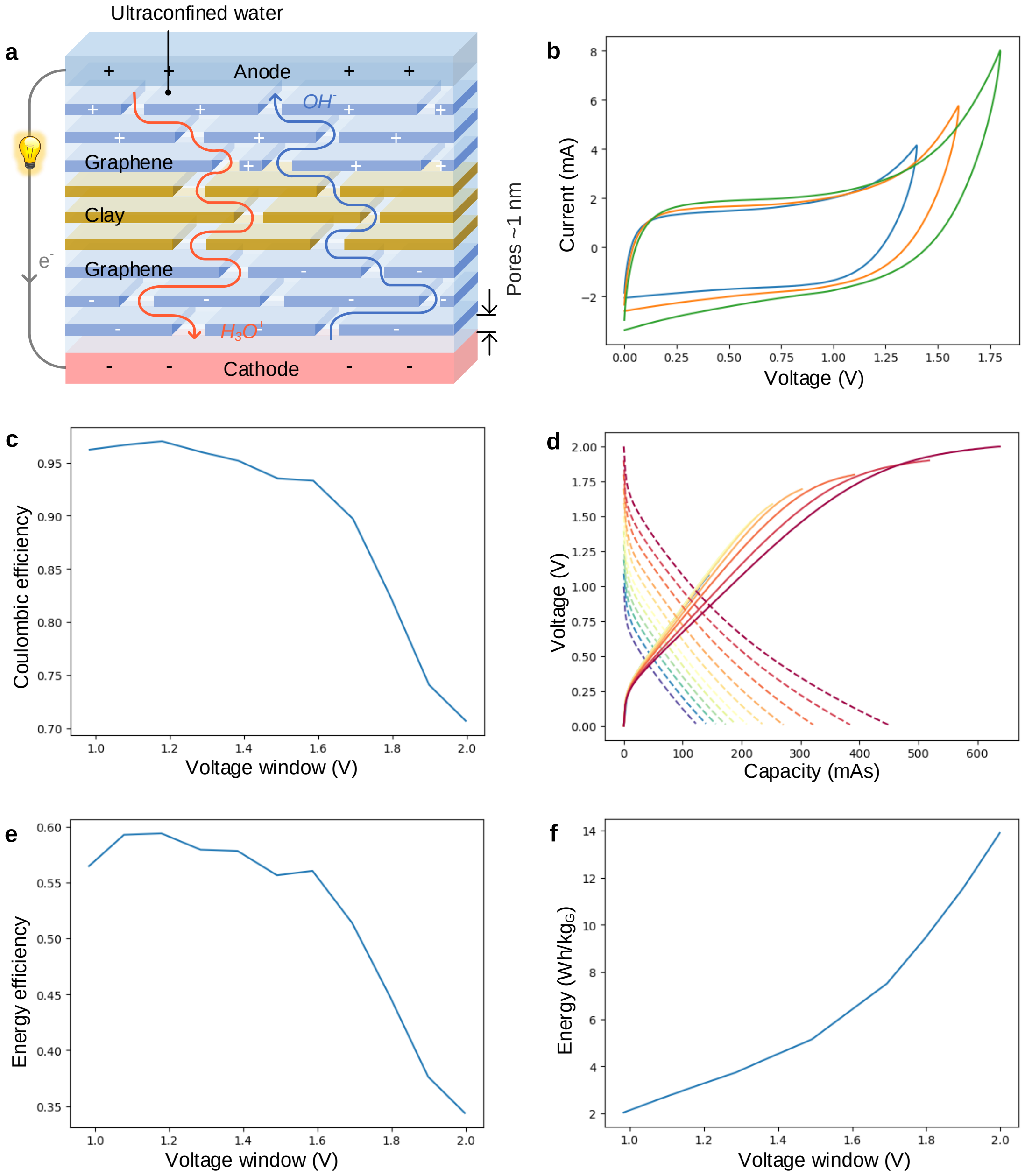

Figure S26. Test for the hydrogen evolution reaction (HER). (a) Schematic representation of the blue capacitor cell. (b, d) Current-voltage characteristics and charge-discharge curves at different cut-off voltages. (c, e, f) Coulombic efficiency, energy efficiency, and specific energy of the cell as functions of the working voltage window. The trade-off voltage is around 1.65–1.70 V. All data at 298 ± 1 K. The HER threshold is approximately 1.65 V, which is higher than the 1.23 V of bulk water. Source data are provided as a Source Data file.

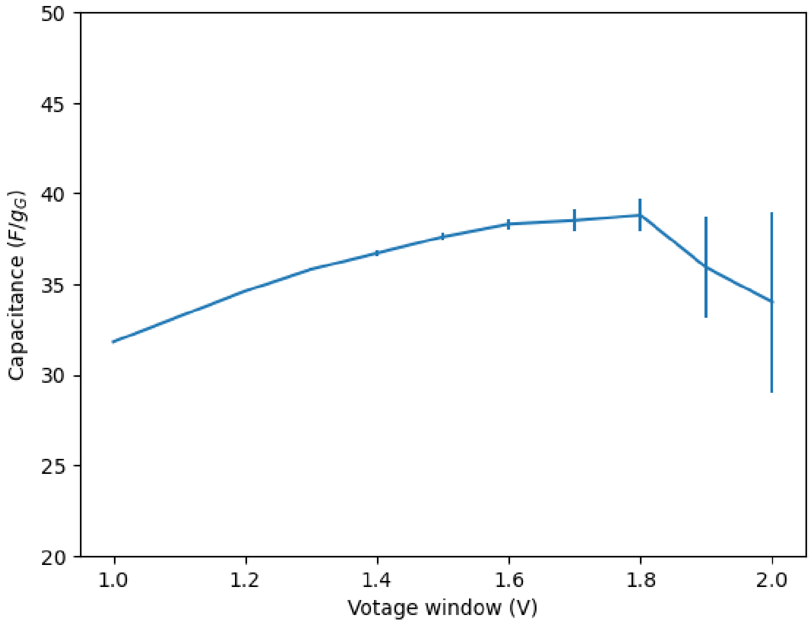

Figure S27. Instability at high voltages. Capacitance recalculated from cycling voltammograms recorded at 0.025 V/s scan rate for voltage windows ranging from 0–1 V and 0–2 V at 298 ± 1 K. Each data point represents 10 repeated cycles; error bars indicate capacitance variation. Source data are provided as a Source Data file.

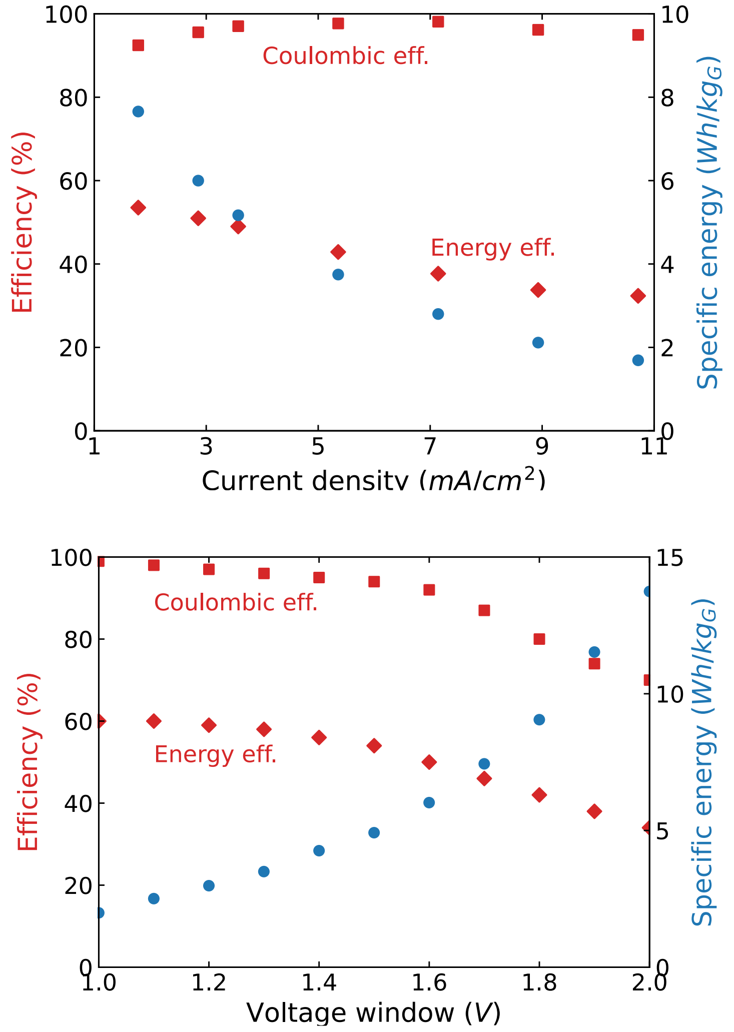

Figure S28. Coulombic and energy efficiencies, as well as specific energy of the blue capacitor, at different current densities (top) and voltage windows (bottom) at 298 ± 1 K. Source data are provided as a Source Data file.

7. SEM, TEM, and STEM imaging

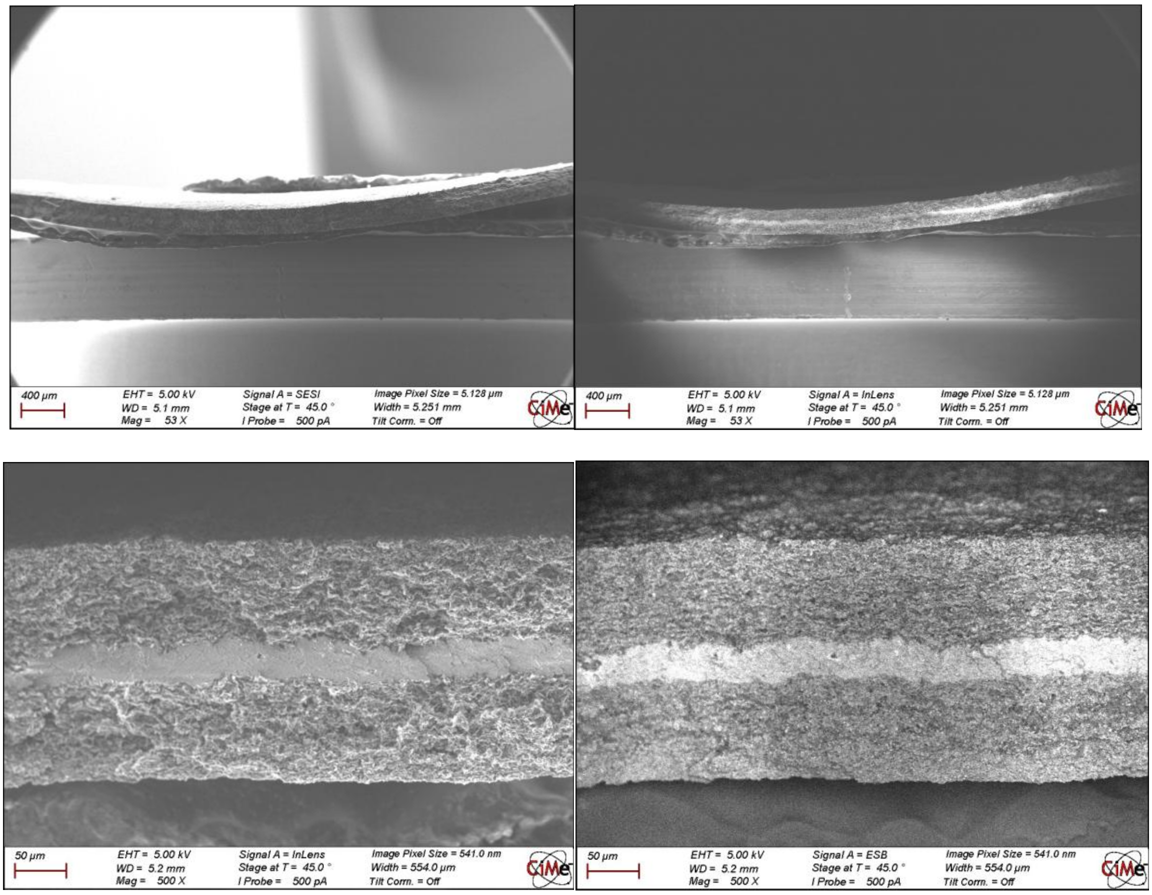

Figure S29. SEM image of the cross-section of dry MEU10 (electrode thickness about 70 µm) at different resolutions (see scalebars and legends). Outer layers: 90% smectite and 10% graphene. Inner layer: 100% smectite.

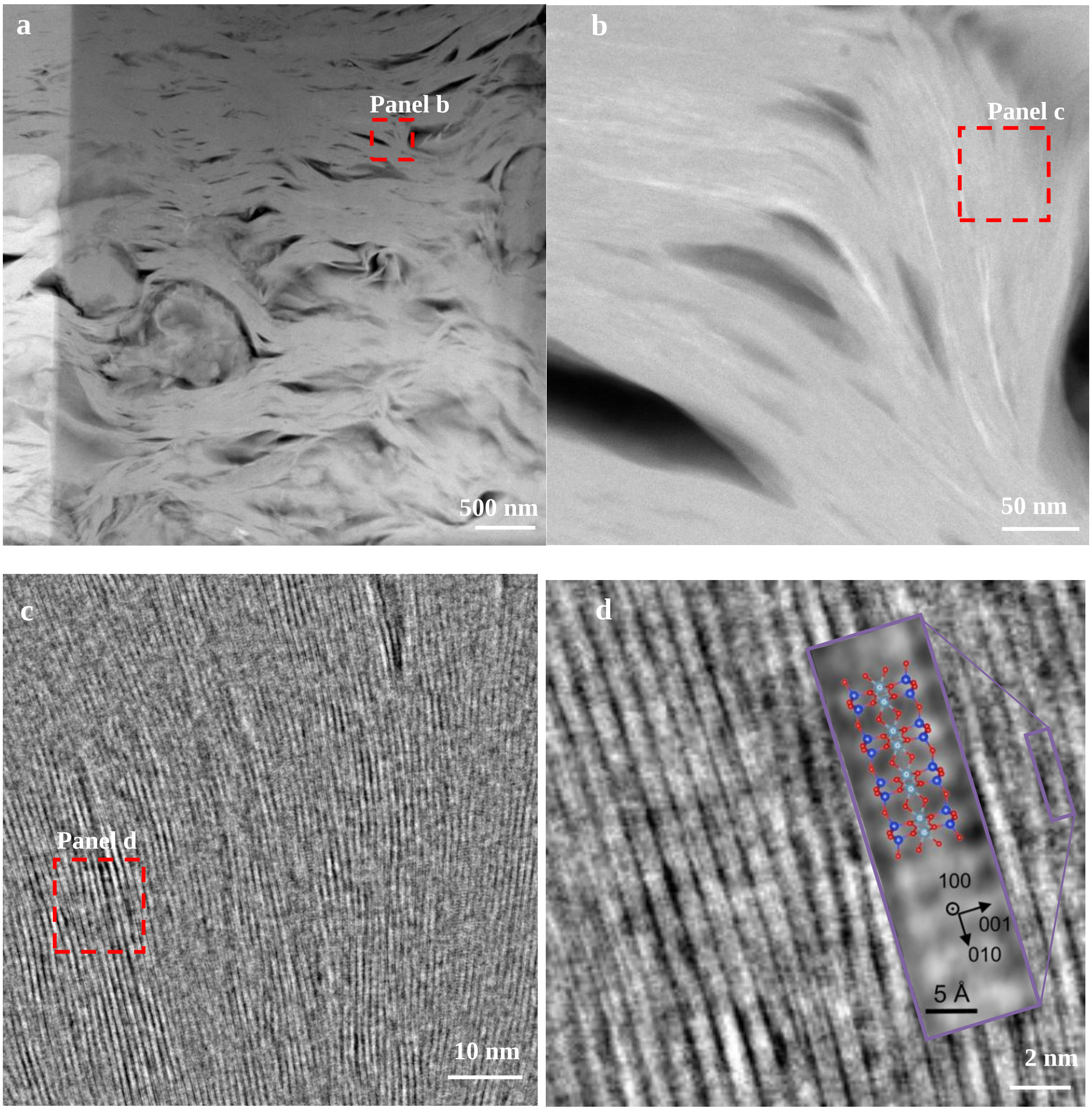

Figure S30. STEM imaging of a cross-section of a smectite membrane at different resolutions (see scale bars). Red squares indicate the part enlarged on the next slide from left to right and top to bottom. (a, b) Annular dark-field (ADF) STEM images at lower magnification. (c,d) Integrated differential phase contrast (iDPC) STEM pictures, used to provide atomic-resolution images at the low-dose condition to prevent degradation of the crystal structure [2]. The inset shows the crystal structure overlapped on the atomic-resolution image. Red, blue, and cyan balls are for O, Si, and Al atoms, respectively.

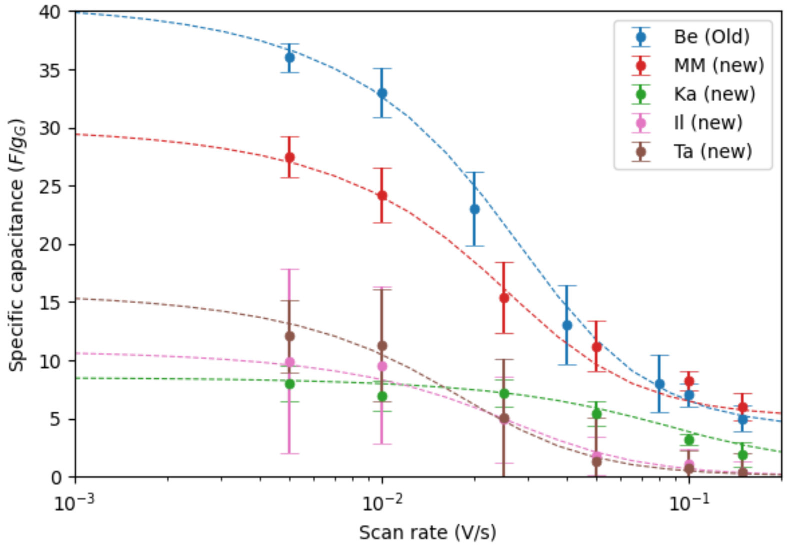

Figure S31. Surface charge dependence test. Specific capacitance of MEUs at different scan rates for five clay types: bentonite (blue), montmorillonite (red), kaolinite (green), illite (pink), and talc (brown) at 298 ± 1 K. Data presented as mean ± SD (n=3 technical replicates). Specific capacitance of the MEUs was estimated from the voltammograms. Source data are provided as a Source Data file.

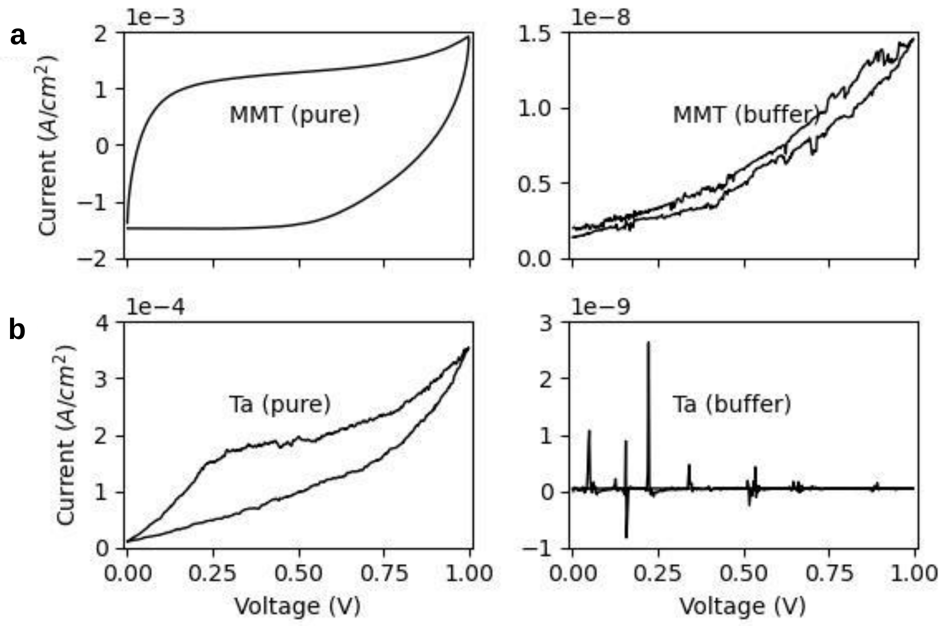

Figure S32. Buffer test. Cyclic voltammograms (CVs) without (left) and with the buffer (right) for montmorillonite (a) and talc-based (b) MEU at 298 ± 1 K. Source data are provided as a Source Data file.

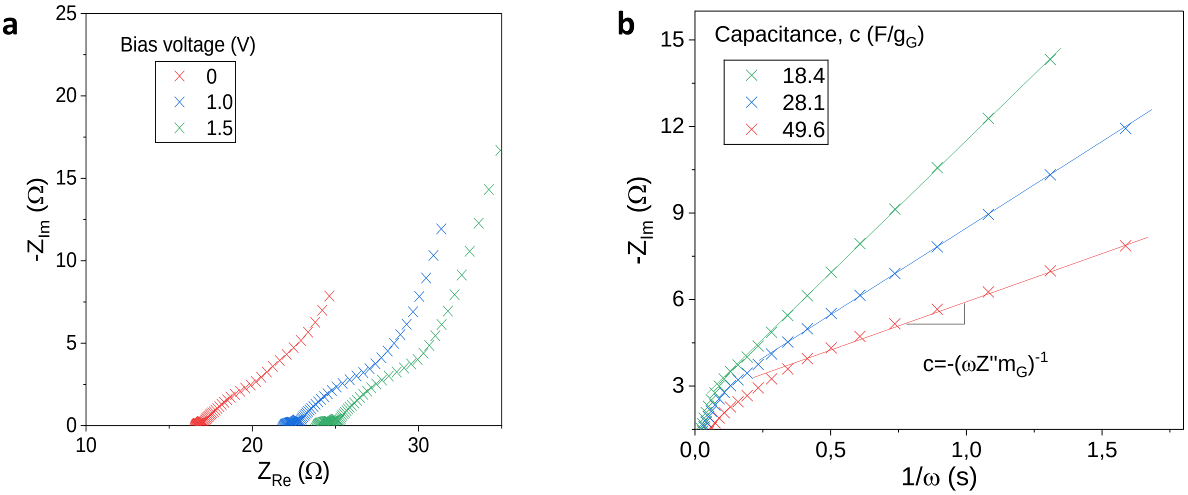

Figure S33. Electrical impedance spectroscopy: bias test. (a) Nyquist plots of in-situ electrical-impedance spectroscopy (EIS) for montmorillonite-based MEU measured at different bias voltages (see legend). (b) Z"(1/ω) graph at low frequencies and capacitances, extracted from slope angles. All data at 298 ± 1 K. Source data are provided as a Source Data file.

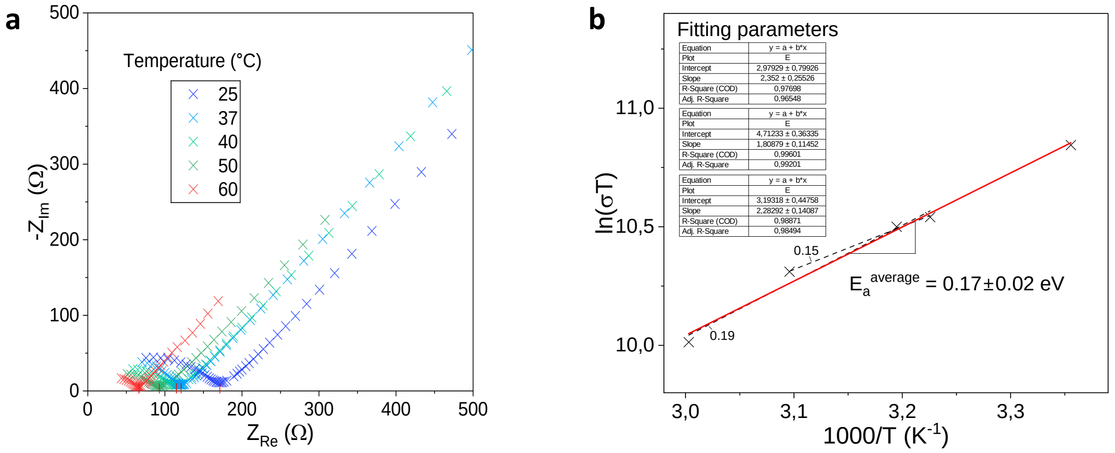

Figure S34. Electrical impedance spectroscopy: temperature test. (a) Nyquist plots of the MEU measured at different temperatures. The vertical sticks show DC resistivity values Z_{dc}. (b) Arrhenius plot of DC conductivity ($\sigma = 1/Z_{dc}$). The number near the curve is the activation energy. Source data are provided as a Source Data file.

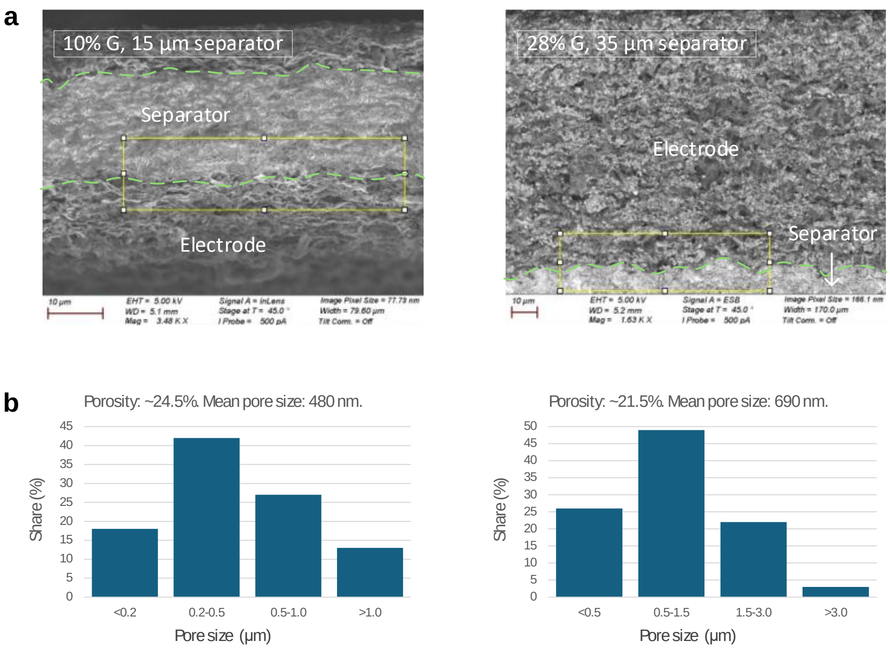

Figure S35. Pore statistics. (a) Example of SEM images of two independently fabricated MEU cross-sections with different concentrations of graphene and different thicknesses of separators. The yellow frame shows a zone processed with ImageJ/Fiji software. (b) Pore size distributions corresponding to the images. Source data are provided as a Source Data file.

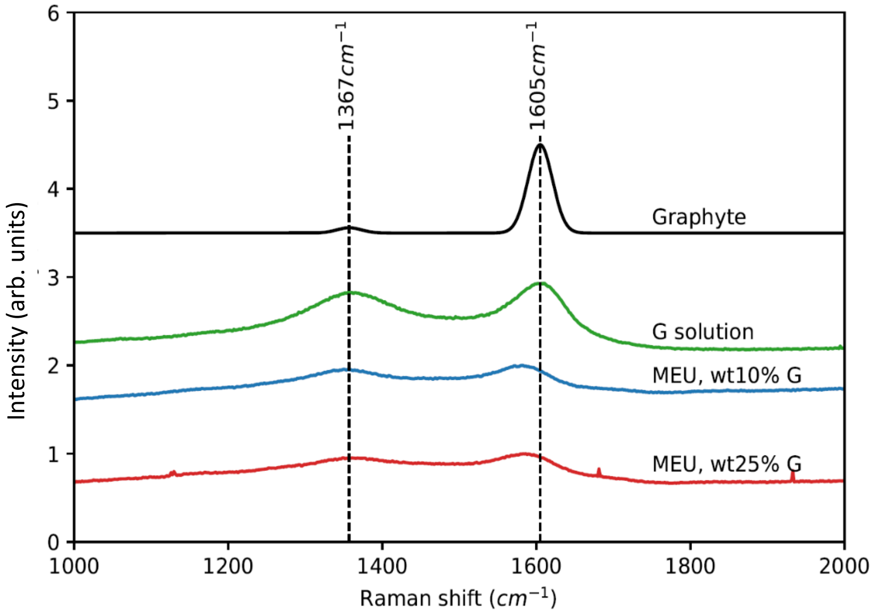

Figure S36. Raman spectra of graphite (black line), graphene solution used for electrodes (green line), and full MEU assemblies with different graphene concentrations as indicated (blue and red lines). Source data are provided as a Source Data file.

Table S3. Comparison of the blue capacitor of this study with other electricity retention technologies.

	Li-ion Batteries	Lead-Acid Batteries	Pumped Hydro Storage	Flow Batteries	Standard Supercapacitors	This study*
Energy/Power density	High/Low	Moder./Low	Low/Moder.	Moder./Low	Moder./High	Moder./High
Lifecycle	500-3000 cycles	200-800 cycles	30+ years	~ 1000 cycles	> 5000 cycles	> 60000 cycles*
Charging time	hours	hours	days	hours	ms to minutes	µs to minutes*
Sustainability	Environmental impact, recycling challenges	Lead pollution, recycling challenges	Low environmental impact	Chemical pollution, habitat disruption	Moderate ecological impact, potential for recyclability	Natural materials, low environmental impact
Safety	Risk of thermal runaway, fire, and explosion	Risk of lead exposure, acid spills	Low risk, but potential for dam breach scenarios	Risk of chemical leakage	Low risk	Low risk
Dependence on raw materials	Rare earth elements (Li, Co, and Ni), subject to supply chain constraints and price fluctuations	Lead and sulfuric acid, subject to price fluctuations	Dependent on water resources, land availability, and regulatory approvals	Vanadium, zinc, iron, or other metal-based electrolytes, subject to supply chain constraints	Carbon-based materials, aluminum, and other metals, generally abundant and readily available	Uses readily and widely available materials, minimal risk of supply chain constraints
Applications	Portable electronics, Electric vehicles, Small-scale storage	Automobile starting and ignition batteries, Small-scale storage	Long-term energy storage from renewable sources	Backup power, short and long-term energy storage	Fluctuating loads (portable devices), Wind and photovoltaic systems peak loads shaving, Defibrillators, Transport braking energy recovery	Same as supercapacitors + Fast-frequency control, Biocompatible devices, Batteries for Mars colonization, Biodegradable batteries.

**Based on lab-scale prototype tests.*

SI References

1. A. Ito, and R. Wagai, Global distribution of clay-size minerals on land surface for biogeochemical and climatological studies. Scientific Data, 4, 170103 (2017).
2. Bosch, E.G.T., Lazic, I., Lazar, S.: Integrated Differential Phase Contrast (iDPC) STEM: a new atomic-resolution STEM technique to image all elements across the periodic table. Microsc. Microanal., 22, 306-307 (2016).
3. I. Bérend et.al., Mechanism of Adsorption and Desorption of Water Vapor by Homoionic Montmorillonites: 2. The Li+, Na+, K+, Rb+ and Cs+-Exchanged Forms, Clays and Clay Minerals, 43, 1995, 324 (1995).
4. J. M. Cases et.al., Mechanism of Adsorption and Desorption of Water Vaporby Homoionic Montmorillonite. 1. The Sodium-Exchanged Form, Langmuir, 8, 2730 (1992).
5. E. Ferrage, Investigation of smectite hydration properties by modeling experimental X-ray diffraction patterns: Part I. Montmorillonite hydration properties, American Mineralogist, 90, 1358 (2005).
6. F. Salles et.al., Ionic Mobility and Hydration Energies in Montmorillonite Clay, J. Phys. Chem. C, 112, 14001 (2008).
7. J. Środoń and Douglas K. McCarty, Surface area and layer charge of smectite from CEC and EGME/H2O-retention measurements, Clays and Clay Minerals, 56, 2008, 155 (2008).
8. Chantel C. Tester et.al., Short- and Long-Range Attractive Forces That Influence the Structure of Montmorillonite Osmotic Hydrates, Langmuir, 32, 12039 (2016).
9. V. Artemov, E. Uykur, P. Kapralov, A. Kiselev, K.J. Stevenson, H. Ouedane, M. Dressel, Anomalously high proton conduction of interfacial water. J. Phys. Chem. Lett. 11, 3623 (2020).
10. V. G. Artemov, Dynamical conductivity of confined water, Meas. Sci. Technol., 28, 014013 (2017).
